# Systemic lupus of pediatric onset in Afro-Caribbean children: a cohort study in the French West Indies and French Guiana

**DOI:** 10.1186/s12969-022-00759-7

**Published:** 2022-11-12

**Authors:** Arthur Felix, Frederique Delion, Benoit Suzon, Elise Martin, Anais Ogrizek, M’hamed Mohamed Sahnoun, Claudia Hospice, Aurelie Armougon, Emma Cuadro, Narcisse Elenga, Moustapha Dramé, Brigitte Bader-Meunier, Christophe Deligny, Yves Hatchuel

**Affiliations:** 1 Department of Pediatrics, Martinique University Hospital, MFME. CHU de La Martinique, La Meynard 97261, Fort-de France, France; 2 Department of Pediatrics, Guadeloupe University Hospital, Pointe-À-Pitre, France; 3 Department of Internal Medicine, Martinique University Hospital, Fort-de France, France; 4 Department of Pediatrics, Andrée Rosemon Hospital, Cayenne, France; 5 Department of Pediatrics, Centre Hospitalier de L’ouest Guyanais, St-Laurent-du-Maroni, France; 6 Department of Clinical Research and Innovation, Martinique University Hospital, Fort-de-France, France; 7 Department of Pediatric Rheumatology, Necker Hospital, Paris, France

**Keywords:** Pediatric systemic lupus, Lupus, Lupus nephritis, Autoimmune disease, Afro-Caribbean

## Abstract

**Background:**

Systemic diseases of pediatric onset are more frequent in the Afro-Caribbean population. We performed a study of patients followed in the French overseas departments of America (FOAD) for pediatric systemic lupus erythematosus (pSLE). The aims were to describe the clinical and biological specificities during childhood in this population.

**Methods:**

A retrospective study was conducted between January 2000 and September 2021. Patients with pSLE were identified from multiple sources: computerized hospital archives, registry of referring pediatricians, adult specialists in internal medicine and the French National Registry for rare diseases. We studied SLE with pediatric onset defined by international criteria.

**Results:**

Overall, 2148 patients were identified, of whom 54 were included. The average follow-up was 8.3 years (range: 0.3—25 years). We observed an increase in new diagnoses over time. At onset, pSLE patients had a median of 10 SLICC criteria (range: 4–12), and the median EULAR/ACR 2019 score was 38 (12—54). At onset, one third of patients had renal involvement, 15% had neurolupus and 41% cardiac involvement. During childhood, 54% had renal involvement, and 26% suffered from neurolupus. Patients suffered a median of 3 flares during childhood, and 26% had more than 5 flares. Patients with younger age at onset had worse outcomes than those who were older at diagnosis, i.e., more flares (median 5, *p* = 0.02) and requiring an average of 4 background therapies (*p* = 0.04).

**Conclusion:**

The outcomes of Afro-Caribbean patients were similar to those in Western population, but with worse disease activity at onset. Further studies should be performed to identify the genetic and environmental factors in this population.

**Supplementary Information:**

The online version contains supplementary material available at 10.1186/s12969-022-00759-7.

## Key messages

1. Largest cohort of pediatric patients with pSLE of Afro-Caribbean origin
2. Higher incidence and increase throughout the years
3. Worst disease activity at onset and outcomes similar to Western Countries

## Introduction

Pediatric systemic lupus erythematosus (pSLE) is more frequent in the Afro-Caribbean population. It has been less widely studied in this population in France, due to ethical issues in recruiting patients based on ethnic origin, and in North America because these populations have less access to care [1]. The French national epidemiological databases compiling national health data (namely the SNIIRAM and PMSI) have estimated the incidence and prevalence of systemic lupus in France [2]. In 2010, it was 41/100,000, and there was an excess prevalence of Lupus in the French overseas departments of America (FOAD) (94/100 k in Guadeloupe and 127/100 k in Martinique [2]). The FOAD (Martinique, Guadeloupe, French Guyana, Saint-Martin, Saint-Barthelemy) have a combined population of approximately 330,000 children. As in mainland France, the health system is free and universal, and there are 2 university hospitals and a regional competence center approved by the French Ministry of Health. Although there are no official ethnic statistics, because collecting ethnic origin is prohibited by French law, a large majority of patients are of black African descent [3] (> 90%). Few data are available on the prevalence of pSLE in the Afro-Caribbean population. In one study based on national data, patients followed for lupus in the 0–18 year age group represented 2% of prevalent cases in France [2], but this proportion reached up to 10–15% [4] in other western countries, and up to 20% in the UK [5]. Pediatric SLE seems to be associated with worse outcomes once the child reaches adulthood, but outcomes during childhood and puberty have been less widely described [4, 5]. The onset of the disease is generally during adolescence, and is uncommon before the age of ten [5].Girls are more often affected than boys, and the female predominance increases significantly with age [6]. pSLE seems to be characterized by greater severity than adult disease, notably due to a higher frequency of severe renal and hematological impairment, more disease flares, infections, and neuropsychiatric manifestations [4, 7]. Patients with pSLE continue to have twofold higher mortality than adults [5, 7]. The consistently high prevalence of lupus in the Afro-Caribbean population suggests that genetic factors are likely to underpin the high risk in this group, and the contribution of environmental factors to this group of diseases is not known. The objective of this study was to retrospectively describe a population of patients from FOAD followed for pSLE between 2000 and 2021, to describe the characteristics of the population at diagnosis, during pubertal development and in adulthood, as well as the clinical and biological features specific to this population. The findings are reported in accordance with the STROBE methodology [8].

## Methods

This was a retrospective study conducted on the period from 01/01/2000 and 01/09/2021. We cross-referenced various sources of patient identification, to ensure exhaustive identification of eligible patients. The list of centers referenced as providing medical care to pSLE patients was obtained via the local registry for rare systemic and autoinflammatory diseases. In each referenced center in every FOAD, we searched the registry of pediatric patients followed up by a referring pediatrician and extracted the list of patients from hospital archives from 2000 to 2021 with a diagnosis of lupus. We used broad search criteria to ensure we did not overlook any patients, including the keywords arthritis, nephritis, pericarditis, non-infectious seritis, and cutaneous lupus. We also searched the registry of adult patients with pediatric onset followed by a reference specialist in internal medicine, and the list of patients registered in the national registry (BAMARA, French National Registry for Rare Diseases, which also includes adult patients). Subsequently, the lists of patients were analyzed according to the relevance of the diagnoses and inclusion criteria to eliminate duplicates. Data collection was completed by consulting follow-up and hospitalization reports available in the patients’ medical files. The duration of the follow-up was determined as the time from the date of diagnosis to the date of last contact. Patients were categorized into groups according to their department of origin, and not according to the place where they were treated.

The variables collected are shown in Table 1 supplementary. The diagnostic criteria for pSLE were defined according to the SLICC and the EULAR/ACR2019 classification [9, 10]. We also calculated damage index based on the SLICC/ACR Damage Index (SDI) [11].Anti-nuclear antibodies (ANA) were considered positive if greater than or equal to 1/320, for both girls and boys. A clinical flare was described as clinical symptoms of the disease combined with biological abnormalities requiring a change in background therapy or steroid pulse (> 1 mg/kg/day). Neurolupus was defined according to international criteria [12, 13]. Lupus-related renal impairment was defined according to the international classification [14]. Antiphospholipid syndrome was defined according to international guidelines [15]. Patients present for a short stay (vacation) in one of the territories and followed in another center were excluded from the analysis. Quantitative data are described as mean ± standard deviation or median and range, as appropriate. Qualitative data are described as number and percentage. Proportions were compared using the chi square or Fisher’s exact test as appropriate. All statistical analyses were performed using Stata software. A *P*-value of < 0.05 was considered statistically significant. The Institutional Review Board of the University Hospital of Martinique approved the study under the number 2021/116.

## Results

**Fig. 1:**
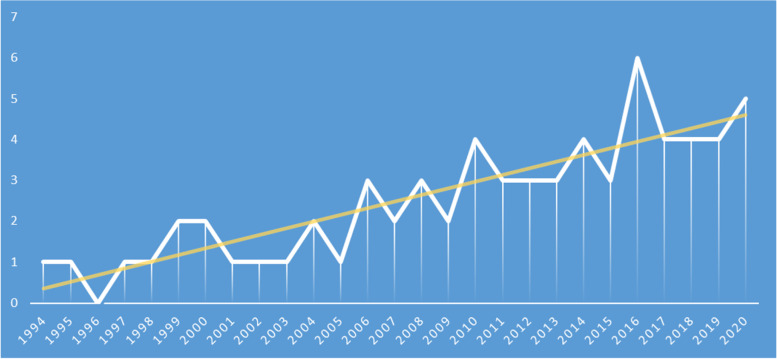
Accrual and temporal distribution of pediatric onset systemic lupus. Number of new patients diagnosed over time according to year of diagnosis. The orange line represents the linear trend

Between 01/01/2000 and 01/09/2021, 2 148 patients were identified, of whom 54 were included in the final analysis (Fig. 1 supplementary). There was a family history of autoimmune disease in 30% of these cases (17/54). The average follow-up duration was 8.3 years, the median was 7 years (range: 0.3 – 25.0 years), and the median age at the date of last follow-up was 21.2 years (range: 14.0—36.7). Follow-up information was available for 27 patients (50%) beyond the age of 18 years. We observed an increase in the number of new diagnoses over the study period (Fig. 1).

The average age at diagnosis was 13.3 years (range: 6–17.4), 80% were girls (43/54) (Table 1). There was no difference in the average age at diagnosis by sex (*p* = 0.27), and the boy/girl ratio did not vary significantly according to age at onset (*p* = 0.31). The average time between symptom onset and diagnosis was 5 months, with a median of 3 months (range: 1 – 48 months). According to the SLICC classification, patients had a median of 10 criteria at onset (range: 4—12). The EULAR/ACR 2019 median score at onset was 38 (range: 12—54). The median erythrocyte sedimentation rate (ESRs) was 90 mm after one hour.

**Table 1:**
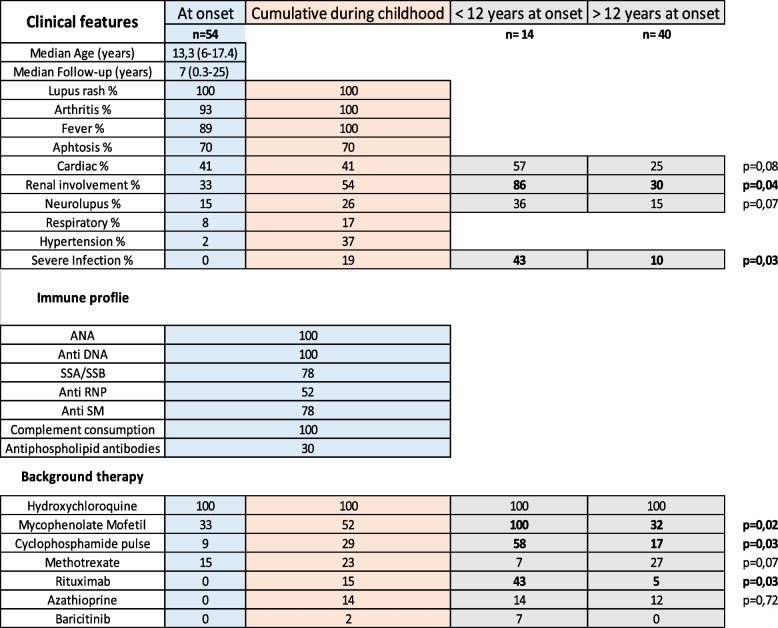
Clinical, biological features, background treatment and outcomes depending on age at onset of patients with pediatric onset systemic lupus

Blue: Features at onset profile and proportion of organ involvement Orange: features during childhood

*ANA* Anti-nuclear antibody

The clinical and biological profiles at onset and during childhood are shown in Table 1. The combination of skin involvement, arthritis and fever was found in 87% of the patients (47/54). Four patients with renal involvement at onset suffered from renal failure requiring dialysis, and 15 underwent kidney biopsy (Table 2 supplementary). Forty-one percent of patients had cardiac involvement, including 5 with myocarditis (9%) (Table 1). Eight patients suffered from neurological involvement at onset (15%), with multiple seizures, psychiatric symptoms, and MRI abnormalities. Five patients at diagnosis had renal, neurological and myocarditis/pericarditis (9%). ANA and anti-DNA antibodies were positive in all patients, followed by anti-Ssa/Ssb (78%), anti-Sm (78%) and anti-RNP (52%).

In total, 29/54 (54%) had renal involvement during childhood, and 11 developed renal involvement during follow-up (Table 1). Overall, 35 types of lupus-related renal involvement were found in 29 patients, and some patients had multiple forms of renal involvement (Table 2 supplementary). For 93% of the patients, renal involvement, if absent at diagnosis, occurred within three years after the diagnosis of pSLE. In our cohort, 14 patients had neurolupus (26%), all of whom had psychiatric symptoms, seizures, and MRI abnormalities, and six patients developed involvement during childhood.

**Fig. 2:**
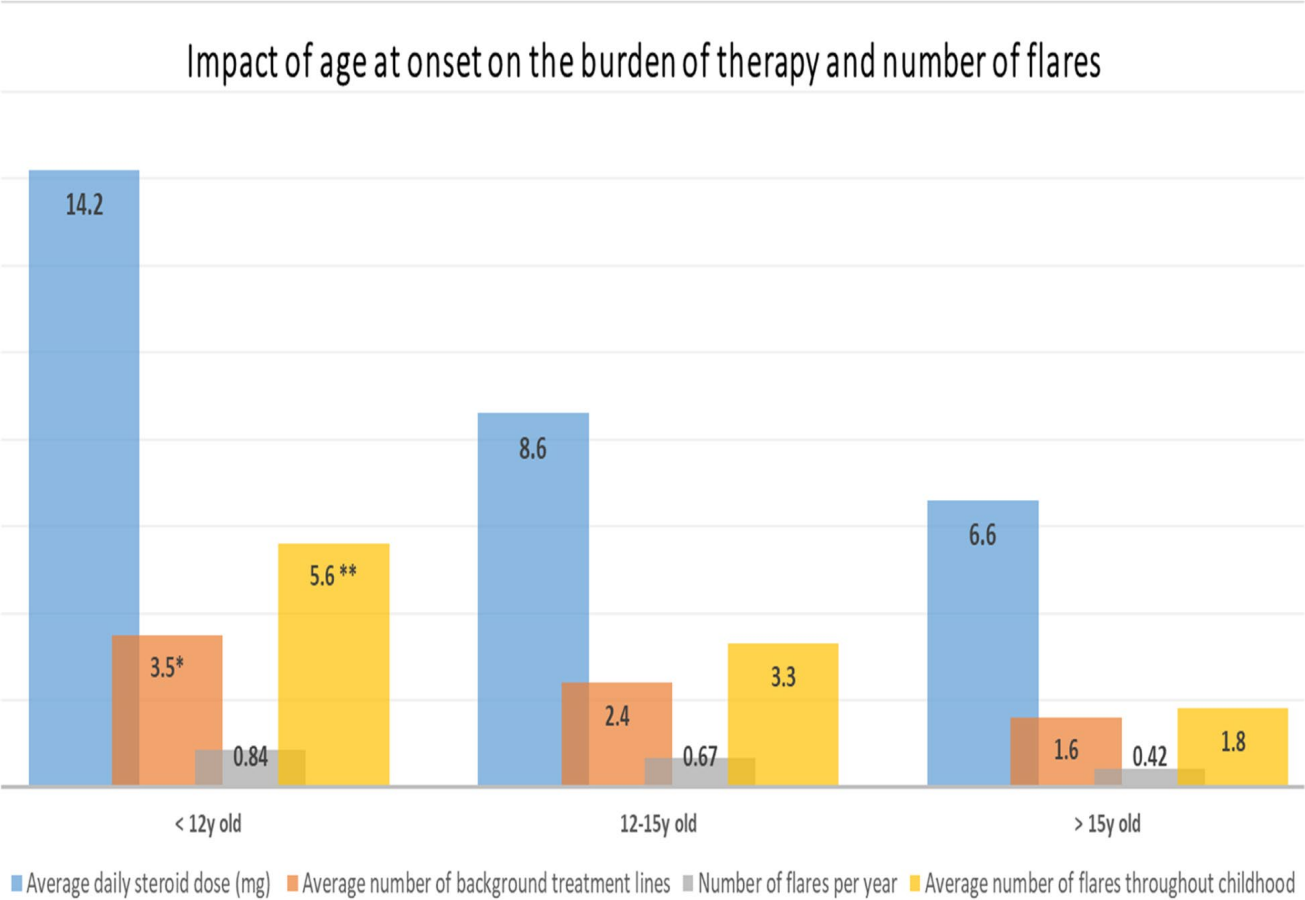
Outcomes during follow up according to age at onset. Average Steroid dose in mg per day. A clinical flare in childhood was described as clinical and laboratory manifestations requiring a change in background therapy or steroid pulse (> 1 mg/kg/day). The number of flares per year during childhood was calculated from the total number of flares and the duration of follow-up before the age of 18 years.*: *P* = 0.04 **P = 0.02

The number of flares during childhood was 3.4 on average, with a median of 3 per patient (range: 1–13) (Fig. 2). Fourteen patients (26%) had more than 5 flares during childhood. The average time that elapsed between 2 flares in our cohort was 15 months (range: 4—30). As background treatment during childhood, only 25% needed hydroxychloroquine (HC). Sixty percent of patients needed two or three lines (mostly HC and methotrexate (MTX) or mycophenolate mofetil (MMF)). Twenty-five percent needed more than three lines of background therapy (HC, MMF, cyclophosphamide pulse, rituximab, azathioprine, plasmapheresis, intravenous immunoglobulin, baricitinib) and suffered from 5 to 13 flares during childhood (Fig. 2). SLICC/ACR Damage Index (SDI) scores were low in our cohort (0 for 52/54 patients).

Sex was not associated with severity of pSLE during childhood (boys vs girls: average number of flares, respectively 3.1 vs 3.4; *p* = 0.42; average steroid dose during childhood, respectively, 9.5 mg vs 10.8 mg; *p* = 0.21). However, at the time of transition to adult care, boys seemed to have better disease control than girls, with median SLEDAI of 3 vs 11 (*p* = 0.12), median ESR after one hour of 5 vs 30 (*p* = 0.03), and median steroid dose at 4 mg vs 10 mg (*p* = 0.04) respectively.

The median age at transition to adult care was 18.2 years (range 16.5–20), median SLEDAI activity score was 12 (range: 0—36), median ESR was 25 (range: 5—79), and the average corticosteroid dose per day was 8.4 mg (range: 0–20). There seemed to be three profiles of disease course in our cohort of pSLE. First, 15% had clinical involvement, which was sometimes florid at diagnosis but very responsive to corticosteroids, requiring only HC and low dose corticosteroids as background treatment. These patients were older at onset (15.3 years old) and had no renal or neurological involvement during follow-up. Second, 26 patients (60%) required two or three background treatment lines, generally for persistent joint, renal, or neurological damage. They did not harbor any specific clinical or biological presentation. Third, 13 patients (25%) had multiple relapses (> 5) during childhood and required significant therapeutic escalation (range: 4–7 lines). These patients were younger at onset (mean age 11 years) and did not harbor a specific immune profile.

Fourteen patients were diagnosed before the age of 12 years old (Table 1 and Fig. 2); half of them had renal or neurological involvement at diagnosis. During childhood follow-up, all but one (93%) had renal and/or neurological involvement, and five had both (35%). These patients had more severe disease, with an average of 5.6 flares during childhood (vs 2.6 for children diagnosed after 12 years old, *p* = 0.02) (Fig. 2). They required an average of four lines of background therapy during childhood (vs 2 for children diagnosed after 12 years old, *p* = 0.04). Half of them required the addition of monoclonal antibodies during childhood (*p* = 0.05). Six patients out of these 14 (43%) had severe infectious events requiring hospitalization, mostly shingles or pneumonia, vs 10% in children diagnosed after 12 years of age (*p* = 0.03). Five patients had dialysis due to end-stage kidney disease, and two of them underwent kidney transplant. The mortality rate was 2%; one patient with severe neurolupus died by drowning.

## Discussion

This retrospective study from the FOAD between 2000 and 2021 describes a cohort of 54 patients suffering from pSLE. This is one of the largest cohorts of pediatric lupus patients of Afro-Caribbean origin. One of the strengths of this work is the multicenter inclusions, with the participation of all the reference pediatricians in the FOAD. Our methodology allowed us to analyze patient therapies by consulting the registries of referring physicians and examining computerized hospital archives in each center using broad inclusion criteria. This explains why, from the original, wide selection of 2148 patients identified, only 54 patients with a diagnosis of pSLE were finally included. This methodology ensured exhaustive identification of patients, with cross-checking of multiple data sources to minimize the loss of patients and data. It should be noted that there is potential for recruitment bias in the hospital computer archives, because they only index patients requiring hospitalization. Nevertheless, most patients with pSLE usually have a short hospitalization at the initial phase. There was also an imbalanced distribution across the departments of origin, with most of the patients registered coming from Martinique (59%). This can be at least partly explained by the fact that the regional competence center for childhood autoimmune diseases is based in Martinique. A second potential explanation is a disparity in identification of patients by the computerized archives system in Guadeloupe.

For pSLE, with a reported incidence of 0.3—0.9 per 100,000 children per year [16], and a total population of 330,000 children aged under 18 in Martinique, Guadeloupe and French Guyana combined, the expected number of pediatric lupus patients over the study period would have been between 21 and 62. We found 54 patients with pSLE, which is in line with the higher frequency of pSLE and adult SLE reported among the Afro-Caribbean populations in Martinique/Guadeloupe [2, 17–19]. The prevalence in our study was likely underestimated because of the memory bias inherent to retrospective studies, and a defect in the computerized hospital archives of one center in Guadeloupe. Since the populations of Martinique and Guadeloupe are similar in number, ethnic original, and environmental exposures, incidence in both departments would be expected to be approximately the same. Despite these limitations, which likely resulted in underestimation of the true number of cases, our cohort nevertheless found a high prevalence of pSLE, notably higher than previously reported in European countries or the Pacific islands [16, 20, 21]. To the best of our knowledge, there is no high-quality study of SLE incidence in cohorts of Afro-Caribbean descent. In one study in a population of US Medicaid beneficiaries (of whom 40% were African American), the authors found an incidence of 2.2 per year per 100,000 children [22], which seems consistent with our results. Similarly, in a study from Northern Italy, Tsioni et al. reported an annual incidence rate of 2.0 (95%CI 0.9–3.8) per 100,000 individuals overall, and of 3.8 (95%CI 0.5–13.8) in children [23].

Our study shows an increase in the number of pSLE over the years in FOAD. Given that cross-checking found 86% agreement between the physician registry and computer records, potentially lost data likely did not significantly affect the trend towards increasing cases over the years but may have underestimated the true number. Moreover, this was observed for 2010–2020, for which memory bias should be at its lowest. The genetic factors and other prominent environmental factor(s) explaining this increasing trend deserve to be explored [24, 25]. This increase is of similar magnitude to that of the two other main chronic autoimmune diseases in children, namely type 1 diabetes, and inflammatory bowel disease (unpublished, personal data). In our cohort, 30% of patients had a family history of SLE, and this strong heritability has previously been described, especially for lupus patients [26]. Conversely, the environmental factors have not been widely studied among the Afro-Caribbean population, especially for those living in the FOAD, and deserves further prospective investigation.

The average length of follow-up was quite long in our study, which enabled detailed study of the progression of these children during childhood. There was an high rate of infectious morbidity requiring hospitalization in our cohort, but none of the children died from infectious complications, which differs from African or non-White patients in emerging countries [27–29]. This infectious risk during childhood has previously been described in pSLE cohorts, with an increased risk in Afro-American populations [30].

The proportion of patients diagnosed after 10 years old was high in our study (85%). This differs from White populations, where the distribution is more balanced [16, 31]. The median duration of symptoms before diagnosis was short (3 months), and mortality in this pSLE cohort was low, with a median follow-up of 7 years; only one patient died from accidental causes (2%). These data show better results than those from emerging countries, and are comparable to statistics from cohorts in Western countries [19, 27, 28]. The children in our cohort had some florid symptoms at onset that were not associated with chronic sequalae (i.e., SDI score). This confirms the clinicians’ impression of greater clinical severity at diagnosis in this population. The classic symptoms at diagnosis were no different from those described in other cohorts, with a triad of febrile skin and joint involvement present in 9 out of 10 patients [27, 28, 31, 32]. The presence of mucosal involvement with oral ulcers (70%) was substantially higher in our cohort than previously described in pSLE or adult cohorts [33]. The proportion of patients with neurological or renal involvement at onset and during childhood corresponds to series described in Western countries, and remains somewhat lower than reports from emerging countries [5, 28, 31]. The proportion of cardiac involvement was significantly higher than in most cohorts described [5, 28, 31]. Despite this, there were no cardiovascular events or chronic, symptomatic impairment of cardiac function in our cohort. This can be explained by the low proportion of patients still being followed up beyond the age of 25 years [34] (16%).These findings nonetheless confirm greater organ involvement at onset and during pSLE [31]. Although the clinical symptoms and findings of laboratory tests are similar to those seen in adults, patients with pSLE tend to have a higher rate of major organ involvement and a more aggressive clinical course [31, 32]. In studies from North America or Africa, ethnic status as Afro-Caribbean seems to be associated with worse prognosis in pSLE. However, the French healthcare system is universal and free of charge, and therefore, the bias related to socioeconomic status and access to healthcare seems to be less significant in our cohort [35, 36]. Thus, with some clinical and biological specificities, patients with pSLE in our cohort had similar rates of organ involvement during childhood and similar overall outcomes to patients described in Western countries [27, 28, 32]. Despite suffering from several flares during childhood, SLICC/ACR Damage Index (SDI) scores were mostly nil in our cohort of patients. Conversely, organ damage associated with childhood flares occurred during adulthood (4 patients on dialysis).

The proportion of patients with anti-DNA, anti-Ssa, anti-RNP, and anti-Sm antibodies was very high in our cohort, and higher than reported in previous studies of White patients, highlighting increased autoantibodies in the population of African descent [37]. This is also consistent with previous reports indicating higher percentages of pediatric patients with anti-Sm and anti-RNP antibodies compared to adult patients [38–40]. The proportion of patients with antiphospholipid antibodies (30%) was also above adult standards [40]. Most of our patients had a marked increase in ESR at onset (80–90), in contrast with a moderately increased CRP level. CRP in pSLE is usually associated with seritis or infection rather than disease flares [41]. In our population, at the age of transition to adult care, boys seemed to have better disease control than girls, which could be explained by the puberty-related increase in testosterone, which has immunosuppressive activity [42].

One of the strengths of this study is the description of the severity of the disease during childhood. Our definition of a flare was based on a marked degree of severity, as opposed to a slight exacerbation of symptoms leading to minor therapeutic adjustment. With a median number of relapses of 3.4 per child, our patients suffer from a substantial flare every 2 years, with almost a third (28%) having more than 5 flares through childhood. In our cohort, patients who were younger at pSLE diagnosis had more severe disease during childhood, and more infectious problems. This has already been described as a criterion for severity for these diseases [43], but not for infectious issues, which can raise the question of the frontier between dysimmunity and immune deficiency in these young patients [44, 45]. Information about pubertal status at onset was poorly and unequally described in the medical records. Since puberty seems to begin earlier in Afro-Caribbean populations [46], we were better able to classify patients according to their age at onset. In addition, puberty starts earlier for girls [46], so classification strictly by pubertal stage would probably have led to a more balanced sex ratio in the prepubertal pSLE group. Organ damage, disease control, morbidity, and mortality in pSLE have previously been linked to risk factors such as young age at diagnosis, male sex, and Afro or Hispanic ethnicity [35, 43] that are not considered in the disease activity score (SLEDAI, EULAR).

## Conclusion

This retrospective cohort of patients with pSLE followed in the FOAD is the largest cohort of Afro-Caribbean children treated in a developed country’s healthcare system. We describe a higher incidence of pSLE in this population, with their clinical and biological characteristics at onset and throughout childhood. The outcomes of Afro-Caribbean children were similar to those of children in Western countries, with worse disease activity at onset. Since our study highlighted a worrisome trend towards a persistent increase in the prevalence of pediatric lupus over time, environmental factors specific to these regions need to be investigated in future prospective studies.

## Supplementary Information

**Additional file 1: Table 1 supplementary.** Description of pediatric systemic lupus patients. **Table 2 supplementary.** Renal involvement at onset and during childhood. **Figure 1 supplementary.** Flowchart of the study population.

## Data Availability

All data generated or analyzed during this study are included in this published article and tables.

## Abbreviations

FOAD: French overseas departments of America
pSLE: Pediatric systemic lupus
ANA: Anti-nuclear antibodies
ESR: Erythrocyte sedimentation rates
HC: Hydroxychloroquine
MTX: Methotrexate
MMF: Mycophenolate mofetil
