# Supplementary material for: Systemic lupus of pediatric onset in Afro-Caribbean children: a cohort study in the French West Indies and French Guiana
Source: Pediatr Rheumatol Online J. 2022 Nov 12;20:95. doi: 10.1186/s12969-022-00759-7 (PMC9652926; doi:10.1186/s12969-022-00759-7)
Supplement: Supplementary file 1 — Additional file 1: Table 1 supplementary. Description of pediatric systemic lupus patients. Table 2 supplementary. Renal involvement at onset and during childhood. Figure 1 supplementary. Flowchart of the study population. [file 12969_2022_759_MOESM1_ESM.docx]

**Systemic lupus of pediatric onset in Afro-Caribbean children: a cohort study in the French West Indies and French Guiana.**

Arthur Felix^1^, Frederique Delion^2^, Benoit Suzon^3^, Elise Martin^4^, Anais Ogrizek^1^, M’hamed Mohamed Sahnoun^5^, Claudia Hospice^1^, Aurelie Armougon^1^, Emma Cuadro^4^, Narcisse Elenga^4^, Moustapha Dramé^6^, Brigitte Bader-Meunier^7^, Christophe Deligny^3^, Yves Hatchuel^1^

1. Department of Pediatrics, Martinique University Hospital, Fort-de France, France
2. Department of Pediatrics, Guadeloupe University Hospital, Pointe-à-Pitre, France
3. Department of Internal Medicine, Martinique University Hospital, Fort-de France, France
4. Department of Pediatrics, Andrée Rosemon Hospital, Cayenne, France
5. Department of Pediatrics, Centre Hospitalier de l’ouest Guyanais, St-Laurent-du-Maroni, France
6. Department of Clinical Research and Innovation, Martinique University Hospital, Fort-de-France, France
7. Department of Pediatric Rheumatology, Necker Hospital, Paris, France.

Corresponding author: Dr Arthur FELIX [Arthur.felix@bluewin.ch](mailto:Arthur.felix@bluewin.ch)

*MFME. CHU de la Martinique La Meynard 97261 Fort-de-France Tel : +596596709349*

*fax: +596596709359*

**Table 1 supplementary: Description of pediatric systemic lupus patients.**

*Delay to diagnosis is in months, steroids dose in mg per day, time between 2 flares is in months, HC: hydroxychloroquine, MMF: mycophenolate mofetil, MTX: methotrexate, CY: cyclophosphamide pulse, AZ: azathioprine. Lupus-related renal impairment was defined according to the international classification*

**

***Table 2 supplementary: Renal involvement at onset and during childhood***

*Lupus-related renal impairment was defined according to the international classification****.***

*
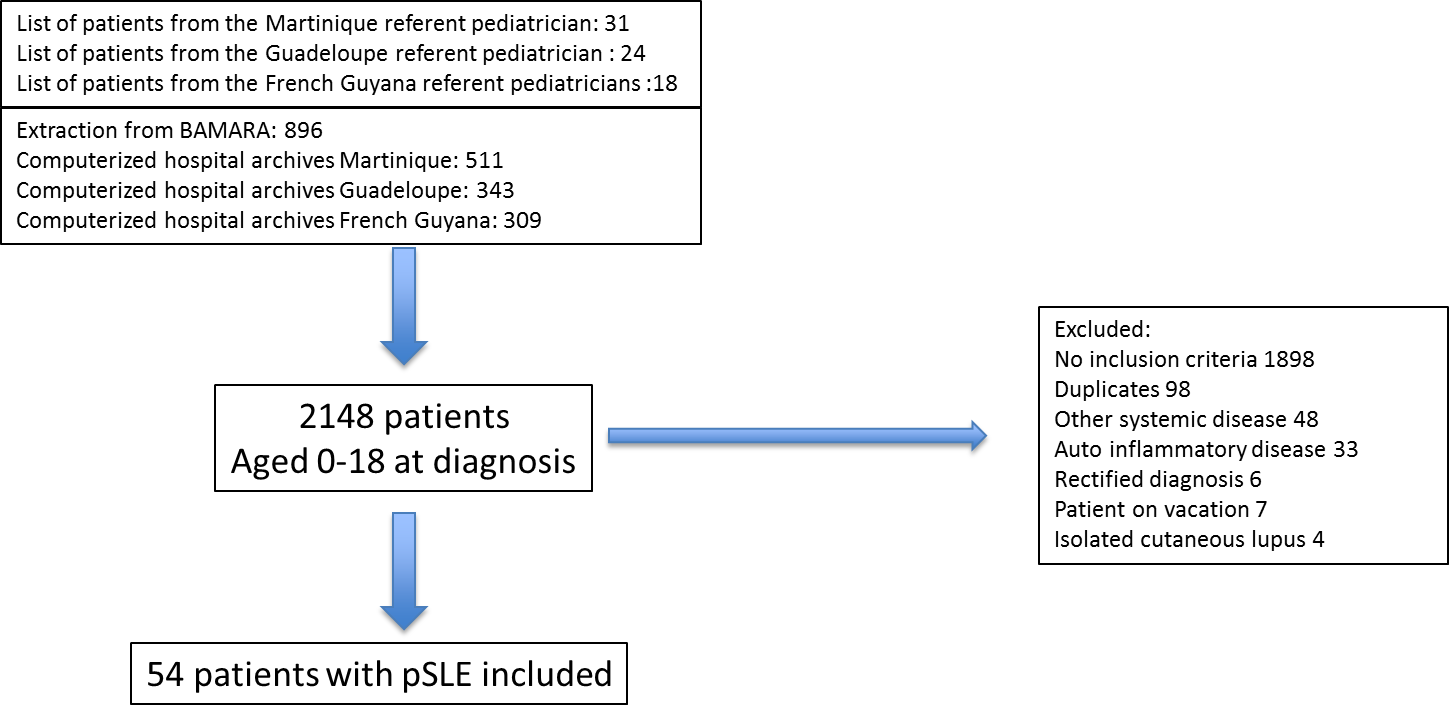
*

***Figure 1 supplementary: Flowchart of the study population.***

*BAMARA is the French national database for rare diseases.*
